# *Chromobacterium Csp_P* biopesticide is toxic to larvae of three *Diabrotica* species including strains resistant to *Bacillus thuringiensis*

**DOI:** 10.1038/s41598-022-22229-6

**Published:** 2022-10-25

**Authors:** Adriano E. Pereira, Man P. Huynh, Kyle J. Paddock, José L. Ramirez, Eric P. Caragata, George Dimopoulos, Hari B. Krishnan, Sharon K. Schneider, Kent S. Shelby, Bruce E. Hibbard

**Affiliations:** 1 Plant Genetics Research Unit, USDA-ARS, Columbia, MO 65211 USA; 2 Division of Plant Sciences and Technology, University of Missouri, Columbia, MO 65211 USA; 3 Department of Plant Protection, Can Tho University, Can Tho, 900000 Vietnam; 4 Crop Bioprotection Research, USDA-ARS, Peoria, IL 61604 USA; 5 Bloomberg School of Public Health, Johns Hopkins University, Baltimore, MD 21205 USA; 6 North Central Agricultural Research Laboratory, USDA-ARS, Brookings, SD 57006 USA; 7 Biological Control Insect Research Laboratory, USDA-ARS, Columbia, MO 65203 USA

**Keywords:** Molecular biology, Plant sciences

## Abstract

The development of new biopesticides to control the western corn rootworm (WCR), *Diabrotica virgifera virgifera* LeConte, is urgent due to resistance evolution to various control methods. We tested an air-dried non-live preparation of *Chromobacterium* species Panama (*Csp_P*), against multiple corn rootworm species, including Bt-resistant and -susceptible WCR strains, northern (NCR, *D. barberi* Smith & Lawrence), and southern corn rootworm (SCR, *D. undecimpunctata howardi* Barber), in diet toxicity assays. Our results documented that *Csp_P* was toxic to all three corn rootworms species based on lethal (LC_50_), effective (EC_50_), and molt inhibition concentration (MIC_50_). In general, toxicity of *Csp_P* was similar among all WCR strains and ~ 3-fold less toxic to NCR and SCR strains. Effective concentration (EC_50_) was also similar among WCR and SCR strains, and 5-7-fold higher in NCR strains. Molt inhibition (MIC_50_) was similar among all corn rootworm strains except NCR diapause strain that was 2.5–6-fold higher when compared to all other strains. There was no apparent cross-resistance between *Csp_P* and any of the currently available Bt proteins. Our results indicate that *Csp_P* formulation was effective at killing multiple corn rootworm strains including Bt-resistant WCR and could be developed as a potential new management tool for WCR control.

## Introduction

The western corn rootworm (WCR), *Diabrotica virgifera virgifera* LeConte, and the northern corn rootworm (NCR), *Diabrotica barberi* Smith & Lawrence, are the most devastating maize pests in the U. S. Corn Belt^1–4^, causing yield losses and control costs of up to $2 billion per year^5^. The southern corn rootworm (SCR), *Diabrotica undecimpunctata howardi* Barber, is a multivoltine and polyphagous pest that feeds on several crops including maize, whereas WCR and NCR are univoltine and feed almost exclusively on maize^1^. Corn rootworm larvae cause significant damage to the maize roots compromising the uptake of water and nutrients, as well as plant stability in heavy infestations^1,6,7^. Although SCR beetles can migrate northward, they usually do not overwinter in most parts of the Corn Belt; thus, damage by SCR larvae is typically not a concern in those areas.

WCR and NCR management tactics rely on crop rotation primarily with soybeans and maize hybrids expressing *Bacillus thuringiensis* (Bt) proteins. The extraordinary ability to rapidly evolve resistance to management tactics has made corn rootworms one of the most challenging maize pests in North America. Recent cases of resistance evolution to Bt maize hybrids in both species^8,9,11–14^ and to RNA interference (RNAi) in WCR^15^ have highlighted the urgent need for insecticides with new modes of action that do not confer cross-resistance to current control methods including Bt and RNAi^16^.

Bacterial insecticidal proteins have been used against insect pests for many years, especially lepidopteran, coleopteran, and dipteran pests, with Bt being the most widely used^17,18^. New insecticidal proteins produced by soil bacteria other than Bt such as *Photorhabdus luminescens*^19^, *Chromobacterium piscinae*^20,21^, *Pseudomonas chlororaphis* and *P. mosselii*^22–25^, *Alkaligenes faecalis*^26,27^ and *Brevibacillus laterosporus*^26,28^, and even those produced by mushrooms from the genus *Pleurotus*^29^ have been explored as future venues for corn rootworm management and resistance management^18^. In addition, recent studies have documented the toxicity of modified or engineered Bt proteins such as Cry6Aa1^30^, CytAa^31^, Cry8Hb^32^, and the first vegetative insecticidal protein Vpb4Da2^33,34^ against WCR larvae. Finding new molecules with distinct modes of action without cross-resistance to current technologies should be the goal for corn rootworm discovery programs, given the pest’s ability to evolve resistance to multiple control tactics^35–39^, with possible multiple resistance mechanisms^9,13,14,40^.

*Chromobacterium* is a Gram-negative soil bacterium that produces insecticidal metabolites documented to be toxic not only to mosquitoes^41,42^, but also to WCR larvae^20^. The *Chromobacterium* species *Panama* (Betaproteobacteria: Neisseriaceae), referred to *Csp_P* from hereafter, was isolated from mosquito vectors *Aedes aegypti* midguts, which were collected in dengue-endemic areas in Panama^41,42^. *Csp_P* has been demonstrated to have high insecticidal activity against the larvae from three mosquito vector species^42,43^. The objective of the present study was to evaluate the susceptibility of multiple WCR strains including Bt-resistant, -susceptible and diapause strains, diapause and non-diapause NCR strains, and an SCR strain, to an air-dried non-live preparation of the *Csp_P*. In 7-d diet overlay toxicity assays, we estimated the lethal concentration (LC_50_) based on survival data, the effective concentration (EC_50_) based on dry weight of surviving larvae, and the molt inhibition concentration (MIC_50_) based on the number of larvae reaching second instar. Our results indicated that *Csp_P* was effective at killing multiple corn rootworm strains including WCR larvae resistant to Bt toxins.

## Results

### Diet assays

**Figure 1:**
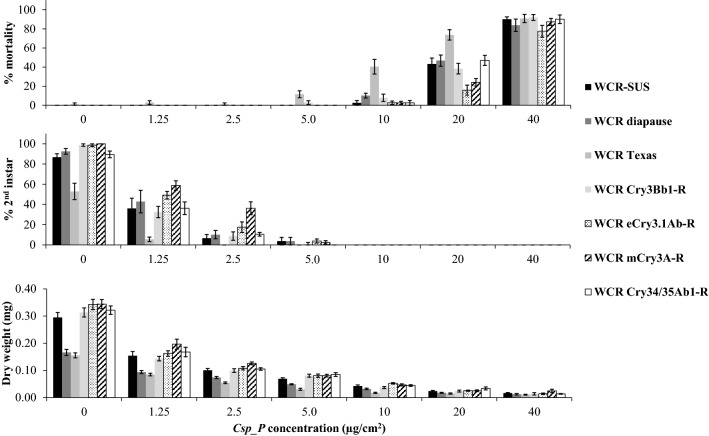
Percent mortality, percent second instar, and dry weight of multiple WCR strains exposed to increasing concentrations (µg/cm^2^) of an air-dried preparation of *Chromobacterium* formulation, in 7-day diet overlay toxicity assays. Each time point in the bars is the average (± SE) of 10 replicates with eight neonate larvae per concentration per replicate.

**Figure 2:**
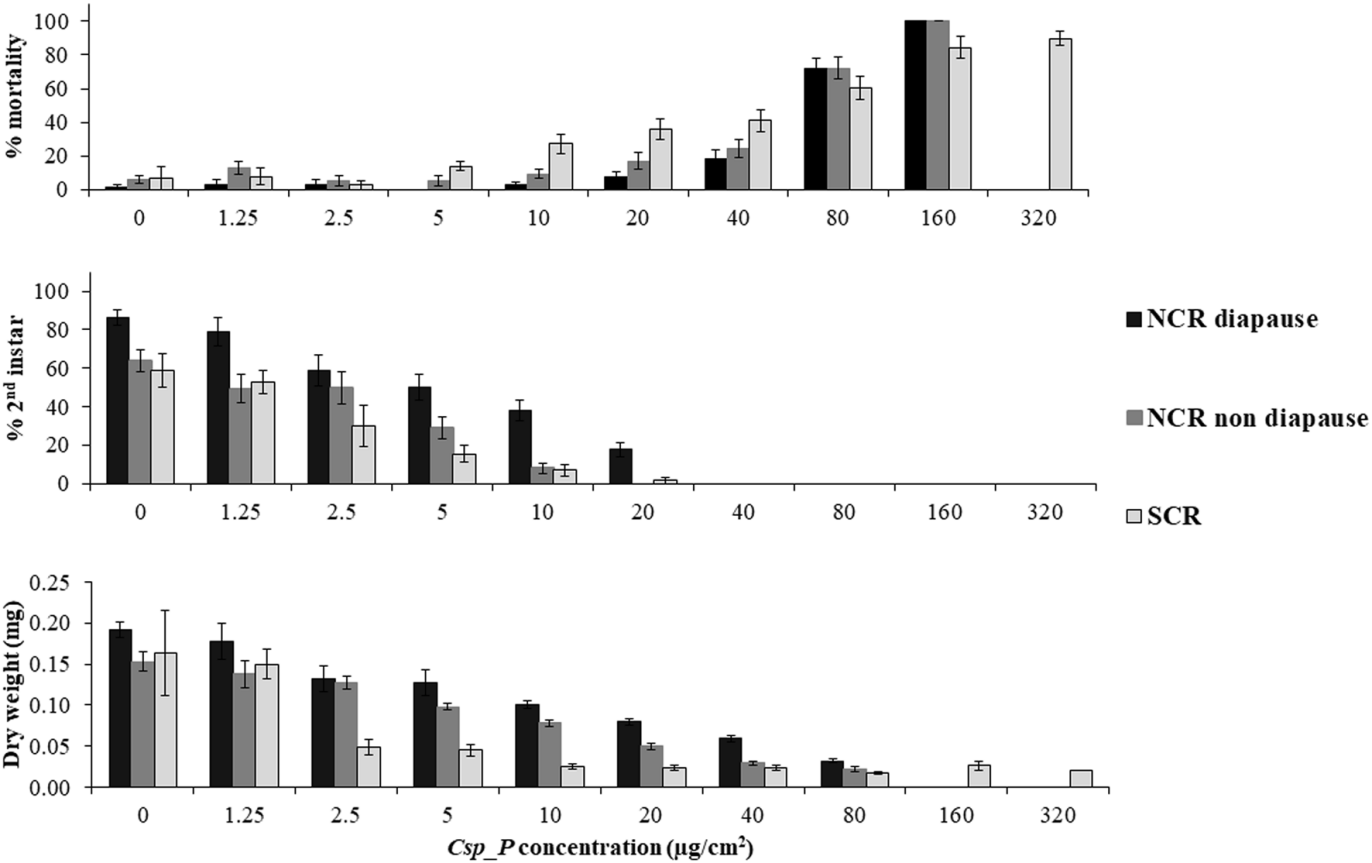
Percent mortality, percent second instar, and dry weight of NCR and SCR strains exposed to increasing concentrations (µg/cm^2^) of an air-dried preparation of *Chromobacterium* formulation, in 7-day diet overlay toxicity assays. Each time point in the bars is the average (± SE) of 10 replicates with eight neonate larvae per concentration per replicate.

After 7 d, the highest concentration of the *Csp_P* preparation used in the assays (40 µg/cm^2^ for WCR, 160 µg/cm^2^ for NCR, and 320 µg/cm^2^ for SCR) killed between 78 and 92% of the WCR larvae across strains (Fig. 1), 100% of the NCR larvae and 90% of the SCR larvae (Fig. 2). The LC_50_ values ranged from 12.9 µg/cm^2^ (WCR Texas) to 61.1 µg/cm^2^ (NCR diapause and non-diapause) (Table 1), the EC_50_ values ranged from 1.30 µg/cm^2^ (WCR eCry3-R and Texas) to ~ 10 µg/cm^2^ (NCR diapause and non-diapause) (Table 1), and the MIC_50_ values ranged from < 1.25 µg/cm^2^ (WCR Texas) to 8.46 µg/cm^2^ (NCR diapause) (Table 1). The WCR eCry-R strain exhibited the highest LC_50_ among all but WCR mCry3A-R strain, with no overlapping 95% confidence intervals (C.I.), whereas WCR Texas exhibited the lowest LC_50_ and MIC_50_ values of all the strains (Table 1). The EC_50_ values were similar among all WCR strains (except for WCR Texas) as were MIC_50_ (Table 1). The NCR strains exhibited the highest LC_50_, EC_50_, and MIC_50_ values, that ranged from 2-fold to 18-fold higher than the WCR strains (Table 1). The SCR strain exhibited higher LC_50_, similar EC_50_, and similar MIC_50_ values than all the WCR strains, except WCR Texas (Table 1).

**Table 1:** Susceptibility (LC_50_, EC_50_, and MIC_50_) of multiple WCR, NCR diapause and non-diapause, and SCR strains to an air-dried preparation of *Chromobacterium*, in 7-day diet overlay toxicity assays.

WCR/NCR strain	N*	Slope (± SE)	LC_50_ (95% CI), µg/cm^2^	EC_50_ (95% CI), µg/cm^2^	MIC_50_ (95% CI), µg/cm^2^	*X*^2^ (d.f.)
WCR SUS	505	5.23 (0.53)	22.1 (20.1–24.3)	1.39 (1.15–1.61)	1.30 (1.06–1.93)	0.55 (4)
WCR mCry3A-R	555	5.48 (0.58)	25.6 (23.4–28.1)	1.61 (0.48–3.07)	2.33 (1.99–2.76)	1.74 (4)
WCR eCry3.1Ab-R	525	4.93 (0.56)	29.1 (26.3–32.4)	1.30 (0.98–1.60)	2.10 (1.69–2.65)	2.73 (4)
WCR Cry3Bb1-R	544	4.09 (0.39)	21.0 (18.8–22.6)	1.58 (1.23–3.91)	1.73 (1.42–2.08)	9.61 (4)
WCR Cry34/35-R	530	5.16 (0.52)	21.5 (19.5–23.7)	1.41 (0.99–1.79)	1.99 (1.73–2.34)	1.69 (4)
WCR diapause	554	3.91 (0.38)	21.6 (19.4–24.2)	1.75 (1.34–2.15)	1.41 (0.89–2.07)	0.83 (4)
WCR Texas	541	3.30 (0.51)	12.9 (11.3–14.8)	1.35 (1.05–3.87)	< 1.25	1.41 (4)
NCR diapause	548	5.19 (1.03)	61.1 (49.8–73.8)	10.2 (7.47–14.0)	8.46 (5.58–13.2)	6.95 (6)
NCR non-diapause	621	5.25 (0.86)	61.1 (45.3–73.7)	9.9 (6.02–16.0)	3.50 (2.16–5.48)	9.57 (6)
SCR	706	1.61 (0.19)	55.9 (37.1–77.1)	2.77 (1.02–7.34)	2.31 (1.34–3.73)	6.23 (6)

*CI* confidence interval, *LC* lethal concentration, *EC* effective concentration, *MIC* molt inhibition concentration.

*Number of insects tested.

### Histologic pathogenesis

**Figure 3:**
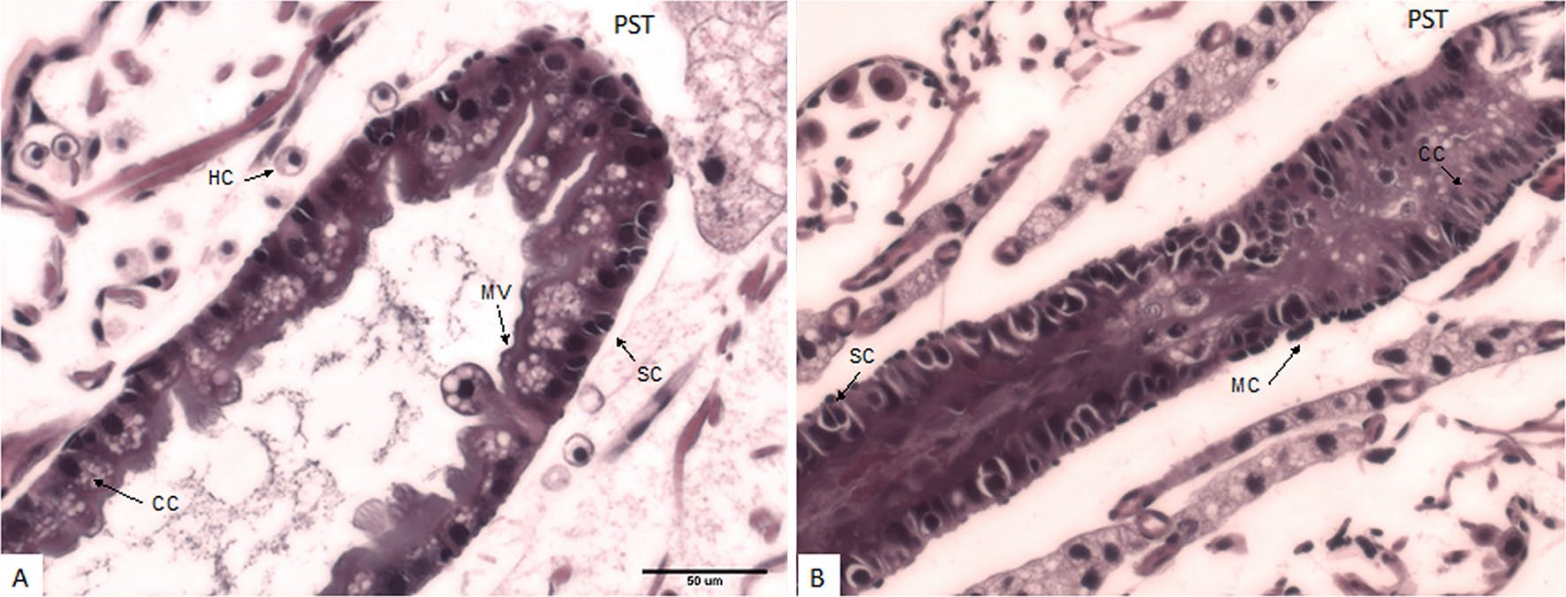
Longitudinal sections of 3-day old western corn rootworm midgut (WCR; *Diabrotica virgifera virgifera* LeConte) larvae reared on artificial diet overlaid with (**A**) sucrose (50 mg/ml) or (**B**) an air-dried *Chromobacterium* preparation at 80 µg/cm^2^. Untreated larvae have intact microvilli (MV) and a single layer of columnar cells (CC) surrounding the gut lumen. Treated larvae exhibit symptoms of muscle constriction from the clustering of muscle cells (MC). The CC are elongated at the posterior (PST) end of the midgut. The anterior portion of the midgut displays dissolved CC and MV and an increased abundance of stem cells (SC). The CC appear multilayered in some places in the treated insects. Cellular debris is visible within the gut lumen.

Normal gut ultrastructure was observed in the WCR feeding for 72 h on a diet supplemented with sucrose alone (Fig. 3A). Columnar cells with an intact brush border membrane lined with microvilli surrounded the gut lumen. However, 72 h of feeding on *Csp_P* significantly affected the gut structure (Fig. 3B). In the treated insects, the microvilli on the apical side of the gut lumen were dissolved. The columnar cells were multilayered as evidenced by cell nuclei along the posterior portion of the midgut. Stem cells could be seen in high numbers toward the anterior portion of the midgut. A dark purple staining observed in the gut lumen of the treated insects may have been commensal bacterial (e.g. bacilli) altered due to the infection by *Csp_P.*

## Discussion

In the present study, we have used diet overlay toxicity assays to evaluate and demonstrate the toxicity of a *Csp_P* formulation against Bt-resistant and susceptible WCR strains, diapause and non-diapause NCR strains, and an SCR strain. We found no evidence of cross-resistance with the WCR Bt-resistant strains, especially with the Cry3Bb1 and Gpp34/Tpp35Ab1 proteins (Table 1; Fig. 1). Cross-resistance between Cry3Bb1, eCry3.1Ab and mCry3A proteins, but not to Gpp34/Tpp35Ab1, has been reported previously^9,10,14,44–47^. The WCR continues to be a challenging maize pest given the recent cases of Bt resistance evolution to Cry3Bb1 and Gpp34/Tpp35Ab1 maize hybrids in North Dakota^13^, Iowa^9^, and Nebraska^14^. In addition, Calles-Torrez et al.^13^ have reported the first case of NCR field-evolved resistance to Bt maize. The EPA has implemented a framework to delay resistance evolution in rootworms^48^. Therefore, in those areas where the WCR have evolved complete or incomplete resistance to Cry3Bb1^10,13,47,49,50^ or to Gpp34/Tpp35Ab1^8,9,11,13,14^, the pyramided maize expressing both Cry3Bb1 and Gpp34/Tpp35Ab1 proteins is expected to perform as a single trait hybrid for WCR control, potentially reducing the time required for resistance evolution to occur and expand throughout a problem region as a consequence^9,51,52^. The addition of a different mode of action, such as the *DvSnf7* double stranded RNA pyramided with Bt in SmartStax Pro for WCR control^53^, or any other bacterial toxin, including but not limited to Bt, could help delay resistance evolution in the field^18^. Based on its toxicity and lack of cross-resistance with Bt proteins reported in this study (Table 1; Fig. 1), the biopesticide *Csp_P* could potentially kill those WCR with homozygous Bt-resistance as well as heterozygotes and delay the development of resistant populations, depending on the concentration and if delivered properly against WCR larvae.

The lack of cross-resistance with the Bt-resistant WCR strains after exposure to *Csp_P* is indicated not only by the similar LC_50_ values we found (except for WCR Texas), but also by the EC_50_ and MIC_50_ values (except for the WCR-SUS, mCry-R, and Texas strains), based on overlapping 95% C.I. values (Table 1). It is important to note that even the lowest concentration of *Csp_P* (1.25 µg/cm^2^) caused significant growth inhibition in all WCR strains, based on lower dry weight and lower percent of 2nd instar larvae when compared to control (Fig. 2). It was not surprising that the field derived WCR from Texas exhibited the lowest LC_50_ and MIC_50_ values when compared to all other WCR strains (Table 1). It is possible that inbreeding depression could explain the strain’s increased susceptibility to *Csp_P*, or it could be related to the genetics of this specific strain collected from maize fields in Texas. It is not unusual that some of the WCR strains exhibit slow growth when compared to other strains. The WCR diapause larvae (and adults) are usually smaller than most diapausing strains (Pereira, personal observation), and this likely explains the lower larval dry weight observed in control (Fig. 1).

We exposed the WCR Texas larvae to *Csp_P* at 80 µg/cm^2^ for 72 h to evaluate histologically and note any symptoms to identify any possible mechanisms involved. It is unclear whether *Csp_P* produces pore-forming toxins, but it appeared to collapse the midgut entirely and dissolve the microvilli in those treated larvae (Fig. 3B), when compared to untreated larvae (Fig. 3A). Such symptoms were not markedly different from those observed in the WCR larvae following Bt exposure^19^. The identity of the insecticidal component(s) of *Csp_P* preparation is still unknown and is currently being identified and characterized.

Sampson et al.^20^ have evaluated the toxicity of purified *C. piscinae* protein against WCR larvae in diet assays, reporting an LC_50_ of ~ 60 µg/cm^2^; this value is two to five-fold higher than the LC_50_ values reported in our study for WCR (Table 1), although they did not report 95% C.I. values in their study. In addition, Sampson et al.^20^ reported less root damage in transformed maize plants expressing *C. piscinae* protein when compared to control plants that did not express *C. piscinae* protein. However, toxicity against SCR was not observed nor were NCR larvae tested in Sampson et al.^20^, as opposed to our study that confirmed activity of *Csp_P* to both species (Table 1).

Even though they are not directly comparable due to different formulations and purity, the LC_50_ values reported in this study for *Csp_P* in WCR larvae were higher than those values reported for purified Bt proteins, using similar diet assays^57^. Depending on the Bt protein, LC_50_ values can vary from 1.60 µg/cm^2^ for Gpp/TppAb1 to 6.39 µg/cm^2^ for mCry3A^57^. These values are approximately 3- to 18-fold lower than those found for *Csp_P* formulation, which was not purified. *Csp_P* formulation was also offered to WCR adults mixed with Cucurbitacin in powder or diluted in sucrose, but no mortality was observed at the concentration tested (data not shown).

In summary, the biopesticide *Chromobacterium Csp_P* was efficient in killing WCR, NCR, and SCR larvae from different strains including WCR resistant to differing Bt toxins with varying levels of susceptibility among the three species, and no cross-resistance with Bt toxins. Resistance management practices, including crop rotation and the use of products that do not confer cross-resistance, should be implemented to avoid the spread of resistance alleles in those areas with resistance. The next steps should focus on determining the corn rootworm active components, testing *Csp_P* as a seed treatment in plant assays for corn rootworm larvae, on selected natural enemies for an initial risk assessment for integrated pest management, and also on non corn rootworm pests to determine if *Csp_P* is a broad-spectrum insecticide.

## Materials and methods

### Insects

Adults of the non-diapausing WCR susceptible and resistant strains were maintained in 30 × 30 × 30 cm BugDorm® cages (BioQuip Products, Rancho Dominguez, CA) at the Plant Genetics Research Unit, USDA/ARS in Columbia, MO. The eggs were collected in 9-cm Petri dishes (Fisher Scientific, Pittsburgh, PA) containing 80-mesh sieved soil and kept at ~ 25 °C for prompt use (colony or assays) or at ~ 9 °C for later use. The rearing techniques are described elsewhere^10,55,56^. The diapausing NCR and WCR strain eggs were received from USDA/ARS in Brookings, SD. SCR eggs were purchased from Crop Characteristics Inc. (Farmington, MN). All egg dishes were kept in a dark chamber at 25 °C until bioassays were initiated.

The WCR colonies resistant to Cry3Bb1 and Gpp34/Tpp35Ab1 are described in Ludwick et al.^57^. These insect strains initially evolved some level of resistance in the field, were crossed to a non-diapausing strain from Brookings, SD, and thereafter were continuously selected on seeds expressing Cry3Bb1 or Gpp34/Tpp35Ab1 at the USDA-ARS facilities in Columbia, MO. The WCR eCry3.1Ab-R strain was generated from a population collected in 2008 by French Agricultural Research Inc. (Lamberton, MN), and has been maintained in the lab for over 40 generations^58^. The mCry3-R strain was generated from beetles collected at three different sites in Missouri in 2006^59^. The WCR Texas strain was generated from a population collected from a mCry3A maize field in 2018 near Hart, Texas, and was suspected to be evolving resistance to mCry3A based on large number of beetles that emerged from the field^60^. Once brought to the lab, the F1 offspring of this colony were crossed with WCR non-diapause beetles from the USDA-ARS in Brookings, SD, to reduce generation time (non-diapause). Root damage data from greenhouse single plant assays indicated no resistance to mCry3A when compared to susceptible strain (data not shown). The strain has been reared on untreated non-Bt corn hybrid (Viking 42–92; Albert Lea Seed, Albert Lea, MN, USA), and is considered susceptible non-diapause field strain.

### Air-dried *Chromobacterium* formulation

A *Csp_P* non-live air-dried preparation was generated by using the method ‘nonlive_1’ in a previously described protocol^43^. In brief, live *Csp_P* were inoculated onto 400 cm^2^ Luria Bertani agar plates and then cultured at 30 °C for 2 days. The plates were covered in Luria Bertani broth and incubated for a further 5 days at room temperature. The liquid phase was then removed, and the *Csp_P* biofilm was collected and air dried before being crushed to a fine powder with a mortar and pestle. The ‘biofilm’ is the bacterial cell growth and debris on the agarose plates after 7 days of growth. It is not a direct growth on the solid agarose surface, since liquid media was added prior to the 7 days growth (see Fig. 1 in Caragata et al.^43^). This preparation contains the insecticidal factor(s) that is/are currently being investigated. This *Csp_P* powder was stored at 4 °C and used in the assays described below.

### Diet assays

The artificial diet used in this research for WCR was an improved diet described by Huynh et al.^61^. The NCR artificial diet was developed specifically for NCR^62^ and the SCR diet was purchased from Frontier Agricultural Sciences (Newark, DE). Rootworm egg sterilization and assays were conducted using methodology similar to that in Ludwick et al.^57^. The *Csp_P* was diluted in sucrose (50 mg/ml, pH 8.8; 99% purity, MP Biomedicals, Inc., Solon, OH) and six concentrations (1.25; 2.5; 5; 10; 20; and 40 µg/cm^2^) plus sucrose alone as control, were prepared in 2-fold dilutions. Sucrose was used in the assays as dilutant because buffers used with Bt proteins (e.g. sodium carbonate/bicarbonate or sodium citrate) usually exhibit some degree of growth inhibition in diet assays, and preliminary assays documented that *Csp_P* diluted in 20 mM of sodium carbonate/bicarbonate buffer, pH 10.0, gave results similar to those for sucrose. For the NCR assays, two additional higher *Csp_P* concentrations were added (80 and 160 µg/cm^2^), since 40 µg/cm^2^ caused ~ 20% mortality; for SCR, one additional concentration (320 µg/cm^2^) was added. The assays were performed in 96-well plates (Costar, Corning Incorporated, Corning, NY; model # 3596). Briefly, 200 µl of artificial diet was poured per well using multi-channel dispenser pipette (Eppendorf Repeater Plus/8 Pipette; No. 022264109, Eppendorf, Hamburg, Germany) and allowed to dry. Twenty microliters of each concentration were dispensed per well using an automatic multi-channel pipette (model # 89134-746, VWR International, Radnor, PA) and allowed to dry in a fume hood for ~ 1 h. One, < 24 h old, neonate larva was transferred per well using a fine paint brush. The plates were sealed with an adhesive 96-well cover (Excel Scientific, Inc., Victorville, CA; Model # TSS-RTQ-100) and one hole was punched per well using a # 0 insect pin for gas exchange. The assays were performed in duplicate, with five replicates (plates) per trial for a total of 10 replicates. Each replicate represented the average measurement of eight insects per concentration. Mortality and the number of larvae that reached the second instar were recorded after 7 days. Surviving larvae from each concentration were collected in 0.5 ml centrifuge tubes containing 70% ethanol. After removing the excess of ethanol, tubes were placed in the oven (Binder Inc., Bohemia, NY; model 115) at 65 °C for at least 48 h, and dry weight was recorded using a Sartorius Cubis ultra-micro scale (Sartorius Corporate, Göttingen, Germany; Model # MSU 6.6S-000-DM) to estimate the EC_50_^63^.

### Histology of exposed and non-exposed WCR larvae

WCR-Texas larvae were exposed to either 80 µg/cm^2^ of *Csp_P* diluted in 50 mg/ml of sucrose or to sucrose alone for 72 h in 96-well plates containing artificial diet, similar as described for diet assays. Larvae were collected after 72 h and placed in 1.5 ml centrifuge tubes containing FAA (50% ethyl alcohol, 5% glacial acetic acid and 10% formaldehyde) for 14 h at 4 °C. Larvae were dehydrated sequentially in a graded ethanol/xylene series and infiltrated with paraffin as described in Kim and Krishnan^64^. Paraffin-embedded larvae were sectioned with a microtome to a thickness of 5 µm and were stained with hematoxylin and eosin. Images of stained larvae sections were acquired to visualize the midgut region and were obtained at 10 × and 40 × with a Leica DM5500B widefield microscope (Leica Microsystems, Buffalo Grove, IL, USA) equipped with a Leica DFC290 color camera.

### Statistical analysis

The mortality (as a percentage) and the percentage of larvae reaching second instar were calculated by dividing the number of surviving or second instar larvae, respectively, by the total number of larvae transferred to the plates for each treatment. The LC_50_ and MIC_50_ data were generated using PROC PROBIT with Distribution = Logistic in SAS 9.4 (SAS Institute, Cary, NC). For LC_50_ and MIC_50_, the number of dead larvae and the number of larvae that reached the second instar, respectively, were used in the analysis to generate the values. Average larval weight was recorded as the pooled larval weight/number of surviving larvae in each treatment. A nonlinear regression model using PROC NLIN in SAS 9.4 was adopted to generate the EC_50_ values by using dry weight data, following the methods described in Marçon et al.^65^. Control mortality in all plates was < 7%.

## Data Availability

All pertinent data are found in the figures and tables. Requests for data and additional information should be submitted to the corresponding author.

## References

[1] Krysan JL, Krysan JL, Miller TA (1986). Introduction: biology, distribution, and identification of pest *Diabrotica*. Methods for the study of pest.

[2] Levine E, Oloumi-Sadeghi H (1991). Management of diabroticite rootworms in corn. Ann. Rev. Entomol..

[3] Gray ME, Sappington TW, Miller NJ, Moeser J, Bohn MO (2009). Adaptation and invasiveness of western corn rootworm: Intensifying research on a worsening pest. Ann. Rev. Entomol..

[4] Meinke LJ (2009). Western corn rootworm (*Diabrotica virgifera virgifera* LeConte) population dynamics. J. Agric. Forest Entomol..

[5] Wechsler S, Smith D (2018). Has resistance taken root in US corn fields? Demand for insect control. Am. J. Agric. Econ..

[6] Hou X, Meinke LJ, Arkebauer TJ (1997). Soil moisture and larval western corn rootworm injury: influence on gas exchange parameters in corn. Agronomy J..

[7] Urías-López MA, Meinke LJ (2001). Influence of western corn rootworm (Coleoptera: Chrysomelidae) larval injury on yield of different types of maize. J. Econ. Entomol..

[8] Gassmann AJ (2016). Evidence of resistance to Cry34/35Ab1 corn by western corn rootworm (Coleoptera: Chrysomelidae): root injury in the field and larval survival in plant-based bioassays. J. Econ. Entomol..

[9] Gassmann, A. J., Shrestha, R. B., Kropf, A. L., St Clair, C. R. & Brenizer, B. D. Field-evolved resistance by western corn rootworm to Cry34/35Ab1 and other *Bacillus thuringiensis* traits in transgenic maize. *P. Manag. Sci*. **76**, 268–276 (2020). 10.1002/ps.5510 31207042

[10] Zukoff, S. N. *et al*. Multiple assays indicate varying levels of cross resistance in Cry3Bb1-selected field populations of the western corn rootworm to mCry3A, eCry3.1Ab, and Cry34/35Ab1. *J. Econ. Entomol*. **109**, 1387–1398 (2016). 10.1093/jee/tow073 27106225

[11] Ludwick DC (2017). Minnesota field population of western corn rootworm (Coleoptera: Chrysomelidae) shows incomplete resistance to Cry34Ab1/Cry35Ab1 and Cry3Bb1. J. Appl. Entomol..

[12] Calles-Torrez V (2018). Transgenic Bt corn, soil insecticide, and insecticidal seed treatment effects on corn rootworm (Coleoptera: Chrysomelidae) beetle emergence, larval feeding injury, and corn yield in North Dakota. J. Econ. Entomol..

[13] Calles-Torrez V (2019). Field-evolved resistance of northern and western corn rootworm (Coleoptera: Chrysomelidae) populations to corn hybrids expressing single and pyramided Cry3Bb1 and Cry34/35Ab1 Bt proteins in North Dakota. J. Econ. Entomol..

[14] Reinders JD, Reinders EE, Robinson EA, French BW, Meinke LJ (2021). Evidence of western corn rootworm (*Diabrotica virgifera virgifera* LeConte) field-evolved resistance to Cry3Bb1 + Cry34/ 35Ab1 maize in Nebraska. Pest Manag. Sci..

[15] Khajuria C (2018). Development and characterization of the first dsRNA-resistant insect population from western corn rootworm *Diabrotica virgifera virgifera* LeConte. PLoS ONE.

[16] Paddock, K. J., M. Robert, C. A., Erb, M. & Hibbard, B. E. Western corn rootworm, plant and microbe interactions: A review and prospects for new management traits. *Insects*. **12**, 171 (2021). 10.3390/insects12020171 PMC7922318 33671118

[17] Wang K, Shu C, Zhang J (2019). Effective bacterial insecticidal proteins against coleopteran pests: A review. Arch. Insect Biochem. Physiol..

[18] Moar WJ, Giddings KS, Narva KE, Nelson ME (2022). Enhancing global food security by using bacterial proteins with improved safety profiles to control insect pests. J. Invertebr. Pathol..

[19] Bowling AJ (2017). Histopathological effects of Bt and TcdA insecticidal proteins on the midgut epithelium of western corn rootworm larvae (*Diabrotica virgifera virgifera*). Toxins..

[20] Sampson K (2017). Discovery of a novel insecticidal protein from *Chromobacterium piscinae*, with activity against western corn rootworm *Diabrotica virgifera virgifera*. J. Invertebr. Pathol..

[21] Zaitseva J (2019). Structure-function characterization of an insecticidal protein GNIP1Aa, a member of an MACPF and β-tripod families. Proc. Natl. Acad. Sci. U.S.A..

[22] Schellenberger U (2016). A selective insecticidal protein from *Pseudomonas* for controlling corn rootworms. Science.

[23] Tabashnik BE (2016). Tips for battling billion-dollar beetles. Science.

[24] Wei JZ (2018). A selective insecticidal protein from *Pseudomonas mosselii* for corn rootworm control. Plant Biotechnol. J..

[25] Boeckman CJ (2019). Characterization of the spectrum of insecticidal activity for IPD072Aa: A protein derived from *Psuedomonas chlororaphis* with activity against *Diabrotica virgifera virgifera* (Coleoptera: Chrysomelidae). J. Econ. Entomol..

[26] Moar WJ, Berry C, Narva KE (2017). The structure/function of new insecticidal proteins and regulatory challenges for commercialization. J. Invertebr. Pathol..

[27] Yalpani N (2017). An *Alcaligenes* strain emulates *Bacillus thuringiensis* producing a binary protein that kills corn rootworm through a mechanism similar to Cry34Ab1/Cry35Ab1. Sci. Rep..

[28] Bowen D (2021). Cry75Aa (Mpp75Aa) Insecticidal proteins for controlling the western corn rootworm, *Diabrotica virgifera virgifera* LeConte (Coleoptera: Chrysomelidae), isolated from the insect-pathogenic bacterium *Brevibacillus laterosporus*. Appl. Environ. Microbiol..

[29] Panevska A (2019). Pore-forming protein complexes from *Pleurotus* mushrooms kill western corn rootworm and Colorado potato beetle through targeting membrane ceramide phosphoethanolamine. Sci. Rep..

[30] Fortea E (2017). Cry6Aa1, a *Bacillus thuringiensis* nematocidal and insecticidal toxin, forms pores in planar lipid bilayers at extremely low concentrations and without the need of proteolytic processing. J. Biol. Chem..

[31] Bravo A (2018). Susceptible and mCry3A resistant corn rootworm larvae killed by a non-hemolytic *Bacillus thuringiensis* Cyt1Aa mutant. Sci. Rep..

[32] Hou J (2019). Engineering of *Bacillus thuringiensis* Cry proteins to enhance the activity against western corn rootworm. Toxins..

[33] Yin Y (2020). A new *Bacillus thuringiensis* protein for western corn rootworm control. PLoS ONE.

[34] Kouadio JL (2021). Structural and functional insights into the first *Bacillus thuringiensis* vegetative insecticidal protein of the Vpb4 fold, active against western corn rootworm. PLoS ONE.

[35] Ball HJ, Weekman GT (1962). Insecticide resistance in the adult western corn rootworm in Nebraska. J. Econ. Entomol..

[36] Meinke LJ, Siegfried BD, Wright RJ, Chandler LD (1998). Adult susceptibility of Nebraska western corn rootworm (Coleoptera: Chrysomelidae) populations to selected insecticides. J. Econ. Entomol..

[37] Levine E, Spencer JL, Isard SA, Onstad DW, Gray ME (2002). Adaptation of the western corn rootworm to crop rotation: evolution of a new strain in response to a management practice. Am. Entomol..

[38] Gassmann AJ, Petzold-Maxwell JL, Keweshan RS, Dunbar MW (2011). Field-evolved resistance to Bt maize by western corn rootworm. PLoS ONE.

[39] Pereira AE (2015). Evidence of field-evolved resistance to bifenthrin in western corn rootworm (*Diabrotica virgifera virgifera* LeConte) populations in western Nebraska and Kansas. PLoS ONE.

[40] Pereira AE, Souza D, Zukoff SN, Meinke LJ, Siegfried BD (2017). Cross-resistance and synergism bioassays suggest multiple mechanisms of pyrethroid resistance in western corn rootworm populations. PLoS ONE.

[41] Ramirez JL (2012). Reciprocal tripartite interactions between the *Aedes aegypti* midgut microbiota, innate immune system and dengue virus influences vector competence. PLoS Negl. Trop. Dis..

[42] Ramirez JL (2014). *Chromobacterium Csp_P* reduces malaria and dengue infection in vector mosquitoes and has entomopathogenic and in vitro anti-pathogen activities. PLoS Pathog..

[43] Caragata, E. P., Otero, L. M., Carlson, J. S., Dizaji, N. B. & Dimopoulos, G. A. Nonlive preparation of *Chromobacterium sp. Panama* (Csp_P) is a highly effective larval mosquito biopesticide. *Appl. Environ. Microbiol*. 86, e00240–20 (2020). 10.1128/AEM.00240-20 PMC7237781 32220845

[44] Gassmann AJ (2014). Field-evolved resistance by western corn rootworm to multiple *Bacillus thuringiensis* toxins in transgenic maize. Proc. Natl. Acad. Sci. USA.

[45] Wangila DS, Gassmann AJ, Petzold-Maxwell JL, French BW, Meinke LJ (2015). Susceptibility of Nebraska western corn rootworm (Coleoptera: Chrysomelidae) populations to Bt corn events. J. Econ. Entomol..

[46] Jakka SRK, Shrestha RB, Gassmann AJ (2016). Broad-spectrum resistance to *Bacillus thuringiensis* toxins by western corn rootworm (*Diabrotica virgifera virgifera*). Sci. Rep..

[47] Reinders JD, Hitt BD, Stroup WW, French BW, Meinke LJ (2018). Spatial variation in western corn rootworm (Coleoptera: Chrysomelidae) susceptibility to Cry3 toxins in Nebraska. PLoS ONE.

[48] EPA. Environmental Protection Agency. EPA’s changes to the Bt corn rootworm resistance management program https://www.regulations.gov/document?D=EPA-HQ-OPP-2014-0805-0094 (2016).

[49] Shrestha RB, Jakka SR, Gassmann AJ (2018). Response of Cry3Bb1-resistant western corn rootworm (Coleoptera: Chrysomelidae) to Bt maize and soil insecticide. J. Appl. Entomol..

[50] Carrière Y (2020). Crop rotation mitigates impacts of corn rootworm resistance to transgenic Bt corn. Proc. Natl. Acad. Sci. USA.

[51] Roush, R. T. Two-toxin strategies for management of insecticidal transgenic crops: can pyramiding succeed where pesticide mixtures have not? *Philos. Trans. R. Soc. Lond., B, Biol. Sci*. 353, 1777–1786 (1998).

[52] Onstad DW, Meinke LJ (2010). Modeling evolution of *Diabrotica virgifera virgifera* (Coleoptera: Chrysomelidae) to transgenic corn with two insecticidal traits. J. Econ. Entomol..

[53] EPA. Environmental Protection Agency. Pesticide registration: EPA registers innovative tool to control corn rootworm. https://www.epa.gov/pesticide-registration/epa-registers-innovative-tool-control-corn-rootworm (2017).

[54] Pereira AE (2019). Comparative susceptibility of western corn rootworm (Coleoptera: Chrysomelidae) neonates to selected insecticides and bt proteins in the presence and absence of feeding stimulants. J. Econ. Entomol..

[55] Meihls LN (2008). Increased survival of western corn rootworm on transgenic corn within three generations of on-plant greenhouse selection. Proc. Natl. Acad. Sci. U.S.A..

[56] Meihls LN, Higdon ML, Ellersieck MR, Tabashnik BE, Hibbard BE (2012). Greenhouse-selected resistance to Cry3Bb1-producing corn in three western corn rootworm populations. PLoS ONE.

[57] Ludwick DC (2018). A new artificial diet for western corn rootworm larvae is compatible with and detects resistance to all current Bt toxins. Sci. Rep..

[58] Frank, D. L., Zukoff, A., Barry, J., Higdon, M. L. & Hibbard, B. E. Development of resistance to eCry3.1Ab-expressing transgenic maize in a laboratory-selected population of western corn rootworm (Coleoptera: Chrysomelidae). *J. Econ. Entomol*. 106, 2506–2513 (2013). 10.1603/ec13148 24498752

[59] Meihls LN, Higdon ML, Ellersieck MR, Hibbard BE (2011). Selection for resistance to mCry3A-expressing transgenic corn in western corn rootworm. J. Econ. Entomol..

[60] Porter, P., Siders, K. & Vyavhare, S. Texas panhandle corn rootworm probably resistant to some Bt corn. Focus in Entomology. https://focusonagriculture.blogspot.com/2018/08/ (2018).

[61] Huynh MP (2019). Development of an improved and accessible diet for western corn rootworm larvae using response surface modeling. Sci. Rep..

[62] Huynh MP (2019). Multidimensional approach to formulating a specialized diet for northern corn rootworm larvae. Sci. Rep..

[63] Siegfried BD, Vaughn TT, Spencer T (2005). Baseline susceptibility of western corn rootworm (Coleoptera: Chrysomelidae) to Cry3Bb1 *Bacillus thuringiensis* toxin. J. Econ. Entomol..

[64] Kim W-S, Krishnan HB (2004). Expression of an 11 kDa methionine-rich delta-zein in transgenic soybean results in the formation of two types of novel protein bodies in transitional cells situated between the vascular tissue and storage parenchyma cells. Plant. Biotechnol. J..

[65] Marçon PC, Young LJ, Steffey KL, Siegfried BD (1999). Baseline susceptibility of European corn borer (Lepidoptera: Crambidae) to *Bacillus thuringiensis* toxins. J. Econ. Entomol..

